# Adaptive Plasticity of Phytochelatin Synthase Under Chromium Stress and Sulfur Availability in *Scenedesmus acutus*

**DOI:** 10.3390/plants15030510

**Published:** 2026-02-06

**Authors:** Michele Ferrari, Matteo Marieschi, Roberta Ruotolo, Radiana Cozza, Anna Torelli

**Affiliations:** 1 Department of Chemistry, Life Sciences and Environmental Sustainability, University of Parma, Viale delle Scienze 11/A, 43124 Parma, Italy; michele.ferrari@unipr.it (M.F.); matteo.marieschi@unipr.it (M.M.); roberta.ruotolo@unipr.it (R.R.); 2 Department of Biology, Ecology and Earth Science, University of Calabria, Ponte P. Bucci, Arcavacata di Rende, 87036 Cosenza, Italy; radiana.cozza@unical.it

**Keywords:** phytochelatin synthase, *Scenedesmus acutus*, sulfur metabolism, chromium tolerance, alternative splicing

## Abstract

Phytochelatin synthases (PCSs) are pivotal enzymes in heavy metal detoxification, yet also implicated in sulfur homeostasis and redox regulation. In this study, we report the molecular and functional characterization of the *PCS* gene from the green alga *Scenedesmus acutus* (*SaPCS*), comparing wild-type and chromium-tolerant strains of this microalga. RT-qPCR, immunoblotting and mass spectrometry analyses revealed that SaPCS expression and protein abundance are primarily regulated by sulfur availability rather than by chromium stress. Two protein isoforms (~70 kDa full-length and ~34 kDa truncated) were detected, both more abundant in the chromium-tolerant strain than the wild-type and responsive to sulfur availability. Furthermore, three alternatively spliced transcript variants (*SaPCSa*, *SaPCSb*, *SaPCSc*) lacking the C-terminal domain coding region but retaining a functional or partially disrupted N-terminal catalytic domain were identified, contributing to the post-transcriptional diversification of PCSs. Mass spectrometry analyses showed negligible phytochelatin production in response to chromium treatment, indicating that detoxification of this metal in *S. acutus* relies mainly on glutathione (GSH) conjugation and the ascorbate–GSH antioxidant cycle. Overall, these results suggest that SaPCS may promote chromium tolerance by modulating sulfur and redox metabolism rather than by driving phytochelatin accumulation, highlighting the remarkable functional plasticity of PCSs in algal stress responses.

## 1. Introduction

Plants are continually exposed to biotic and abiotic stresses that compromise growth, productivity, and survival [1]. To withstand these challenges, plants adopt three main strategies: tolerance, avoidance, and resistance [2], which rely on the coordinated activation of genes and interconnected signaling pathways regulating metabolic networks [3]. PCs are recognized as one of the most effective thiol-based defense systems in plants and algae, allowing for the detoxification of hazardous heavy metals (HMs) [4] through chelation and vacuolar sequestration [5,6].

Among HMs, chromium (Cr) is a widespread heavy metal pollutant in water and soil, representing a worldwide environmental concern, deriving from both natural and anthropogenic sources [7]. While in freshwater Cr(VI) concentrations usually range between 0 and 2 μM (in Cr-rich springs from ultramafic, igneous and metamorphic rocks), Cr(VI) concentrations in effluents of mining, metal works, electroplating, wood and paper processing, leather tanning, and dye industries have been reported to range from ten to a hundred μg/L [8,9]. Although Cr is an essential micro-element for animal metabolism, in plants and algae it is known as a non-necessary and toxic element causing several metabolic damages, such as inhibition of photosynthesis and cell division, oxidative stress, and imbalance of cell nutrition. Upon entering the trophic chain, it is responsible for the biomagnification phenomenon [10]. In its hexavalent state [Cr(VI)], chromium is predominantly present as chromate (CrO_4_^−^) and dichromate (Cr_2_O_4_^2−^) anions, which can readily cross cell membranes via active transport [11,12]. This uptake generally occurs through non-specific anion carriers, mainly exploiting sulfate and phosphate transporters [13,14,15]. The interactions between chromium and sulfur are numerous and complex, beginning with the competition between their chromate and sulfate anions for the same transporters. Once inside the cell, chromium can be reduced and subsequently bound by the reduced sulfur-containing molecules cysteine (Cys) and glutathione (GSH), both produced by the sulfate assimilation pathway [16,17,18]. These thiol-containing molecules play a direct role in chromium defense, both by binding the metal and by undergoing oxidation to reduce the ROS damage generated upon chromium exposure. Conversely, they may also increase Cr toxicity by reducing Cr(VI) to lower oxidation states, thereby enhancing ROS production and generating more genotoxic chromium species [18]. Moreover, Cr(VI) interferes with key enzymes of the sulfate assimilation pathway [16,19,20,21,22], resulting in decreased Cys and methionine synthesis, protein mistranslation and additional effects that resemble sulfur starvation.

One of the main thiol-containing molecules are phytochelatins (PCs), cysteine-rich peptides with the general structure (γ-Glu-Cys)_n_-Gly (*n* = 2–11) [23]. So far, PCs have been found not only in plants (algae, gymnosperm, angiosperm and liverworts), but also in prokaryotes [24] and some animals, which clearly display their presence from the early stages of evolution [25]. PCs are synthesized enzymatically from reduced GSH by phytochelatin synthases (PCSs; EC 2.3.2.15) [26,27], enzymes with γ-glutamylcysteine dipeptidyl transpeptidase activity belonging to the papain-like cysteine peptidase superfamily (PF05023) [23,28,29]. Several physiological studies in plants indicated the role of PCs in the homeostasis and detoxification of toxic metals—such as Pb^2+^, Cd^2+^, Cu^2+^, Ag^+^, Hg^2+^ [30,31,32], including Cr [33,34,35,36,37], and metalloids As^5+^ [38]. Synthesis of PCs has, however, received little attention in algal cells, and no data exist regarding Cr-induced PCs synthesis in microalgae.

Comparative analyses of PCSs in different plant species indicate that these enzymes generally possess a highly conserved N-terminal domain and a more variable C-terminal domain. The N-terminal domain is consistently associated with catalytic activity and contains three conserved residues—Cys56, His162, and Asp180 in *Arabidopsis thaliana* AtPCS1—that form a catalytic triad (C-H-D). Site-directed mutagenesis studies have shown that substitution of any of these residues results in complete loss of AtPCS1 activity [23,28,29]. The first prokaryotic sequences identified were described as half-PCS or PCS-like proteins [29] since they are generally shorter than plant PCSs. Compared to PCS from land plants, these prokaryotic enzymes exhibit distinct N-terminal domain features that affect post-transcriptional regulation and the architecture of the second substrate-binding pocket [39]. These truncated isoforms, which are unable to efficiently extend PC chain length, have been hypothesized to contribute primarily to GSH turnover rather than to PC biosynthesis [39].

The observation that PCSs are expressed even in the absence of heavy metals has led to a wide debate in recent decades about its role in cell homeostasis. It has been suggested that the functions of PCSs extend beyond heavy metal detoxification, encompassing essential metal homeostasis, the metabolism of GS-conjugates, the regulation of GSH levels, and the modulation of immune responses [40,41,42,43,44,45,46,47]. Consistent with this broader functional spectrum, PCSs are constitutively expressed—apparently in an inactive form—even in the absence of metal exposure, and in some microorganisms that express the gene, phytochelatins have never been detected [27,48,49,50,51].

Nowadays, it is known that regulation of *PCS* gene expression extends beyond the canonical gene transcription and post-translational regulation. Both land plants and algae possess more than one PCS gene in their genomes, and several studies have demonstrated that multiple isoforms can be generated through mechanisms such as alternative splicing and intron retention [34,52,53,54].

Despite their prominent role at the basis of the trophic chain and their possible involvement in phytoremediation, microalgae have so far been little studied with regard to the expression and regulation of PCSs.

Unicellular freshwater green algae, including *Scenedesmus acutus*, are primary producers and the first organisms dealing with pollutants to detoxify their effects and/or their entry into the trophic chain. For this reason they are relevant in many phytoremediation studies [55]. Moreover, unicellular green algae are well-established models for investigating adaptive responses to heavy metals due to their ecological relevance, physiological plasticity, and experimental tractability.

Our study aimed to further investigate the interaction between sulfur (S) metabolism and Cr(VI) tolerance and the phenomenon known as Sulfur Enhance Defense (SED) [56]. In this context, *S. acutus*, a freshwater green alga, belonging to the order of Sphaeropleales, represents a particularly valuable system, as a chromium-tolerant strain (Cr-t), obtained in our laboratory through long-term Cr(VI) exposure, offers the opportunity to dissect genetic and metabolic traits underlying differential metal sensitivity [57,58]. In this strain, Cr(VI)-tolerance was found to be heritable and stably fixed in the genome, since the Cr-t population is able to grow in the presence of Cr(VI) even after prolonged culturing in Cr(VI)-free medium [57,58]. Moreover, previous studies have shown that the different Cr(VI) sensitivity observed in this strain may be due to differences in S metabolism [59,60,61,62]. These studies further showed a transient increase in Cr(VI)-tolerance in both the wild-type (wt) and Cr-t strains after a period of S-starvation. After medium renewal following S-starvation, S-replete cells of both strains showed significantly higher sulfur content compared to S-sufficient cells, resulting in enhanced Cys and GSH production [59,60,61,62], with the increase in all these parameters being significantly more pronounced in the Cr-t strain [59,60,61,62]. In the latter, the accumulation of free Cys in S-replete control cells was surprisingly elevated, without, however affecting the negative feedback regulation of the S uptake/assimilation pathway [61,62]. Conversely, when exposed to Cr(VI), their free Cys content was found to be similar to that observed in the wt cells grown under similar conditions [62]. Since Cys homeostasis within the cell has to be tightly regulated, these observations led us to hypothesize that the lack of negative feedback, despite the high Cys content in Cr-t S-replete cells under control conditions, was due to the masking of free Cys by its inclusion in highly Cys-rich molecules, and that the decrease in free Cys content after Cr(VI) exposure was instead due to metal binding. Both these effects could presumably be attributable to PCs, and this prompted us to investigate the role of PCS on Cr(VI)-tolerance.

Despite the important role of PCs in metal tolerance, the molecular basis of PCS regulation in *S. acutus* remains poorly characterized, and the interplay between S nutrition, Cr(VI) tolerance and PCS activity is still largely unexplored.

In the present study, we report the first characterization of the *PCS* gene from *S. acutus* (*SaPCS*) and investigate its transcriptional and translational regulation in response to S availability and Cr(VI) exposure. Both are ROS-producing abiotic stresses affecting one another, and their effects are strictly inter-related [15,59,60,61]. Since Cr(VI) is an anion, it is not generally considered as a target of PCs and, to our knowledge, Cr(VI)-tolerance as a function of S availability has never been related to PCS activity in microalgae. Our studies, taking advantage of the two *S. acutus* strains with different Cr(VI)-tolerance and different S metabolism, were therefore also aimed at evaluating the contribution of SaPCS in Cr(VI)-tolerance and PCs production, either as a chelating agent or as a source of reduced sulfur accumulation capable of conferring a faster cellular defense based on internal thiols. Our data reveal that, even possessing a single *PCS* gene, *S. acutus* can produce multiple SaPCS isoforms, likely generated through proteolytic processing and/or alternative splicing, with differential expression patterns between wt and Cr-t strains, strengthening the hypothesis of SaPCS involvement in the different Cr(VI) tolerance in the two strains. Together, our findings provide new insights into the functional plasticity of PCS in green algae and highlight the complexity of thiol-mediated responses to Cr stress, strengthening the hypothesis of a role for PCSs lacking a C-terminal domain in counteracting ROS-generating abiotic stresses.

## 2. Results and Discussion

### 2.1. Sequence Analysis of PCS from S. acutus

**Figure 1:**
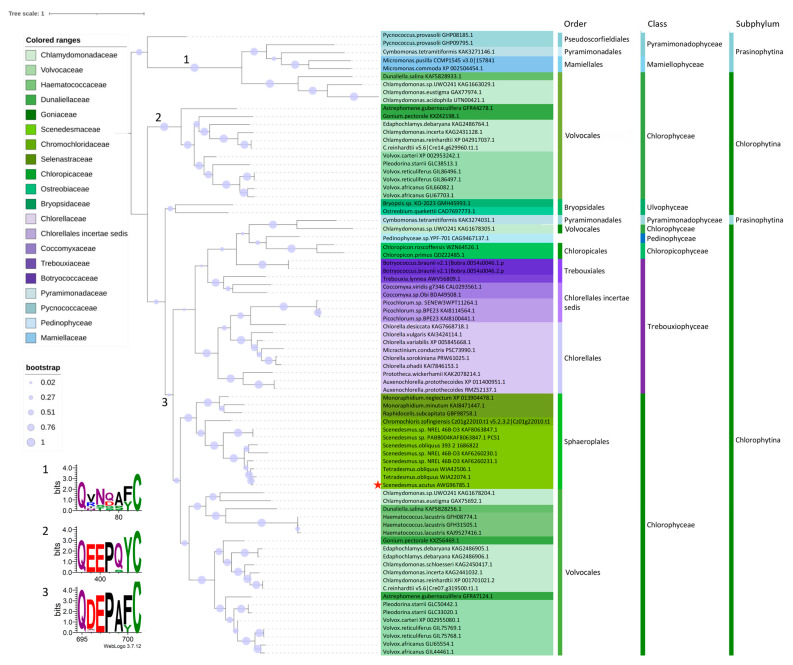
Phylogenetic analysis of green algae PCSs. SaPCS is marked with a red star. WebLogos representing the six amino acids upstream of the conserved cysteine (C) in the catalytic triad for the three principal branches identified in the phylogenetic tree are shown at the bottom left of the figure.

The full-length coding DNA sequence (CDS) of the *SaPCS* gene (GenBank accession N.: MF278027) was obtained by comparing sequences amplified from genomic DNA and retrotranscribed mRNA (cDNA) isolated from a wild-type strain. In silico analysis revealed that the identified full-length CDS (1938 bp) encodes a protein sequence of 645 amino acids with a calculated molecular weight of 70.03 kDa. The SaPCS protein sequence was compared with PCS sequences retrieved from data banks and belonging to different green algae taxa (Table S1). SaPCS contains the N-terminal phytochelatin domain (Pfam accession N.: PF05023), which is highly conserved in all PCS sequences identified, and includes the catalytic triad (C–H–D) (Figure S1). Notwithstanding, although SaPCS possesses a long C-terminal region containing 11 residues of Cys, the canonical Phytochelatin_C domain (PF09328) is not recognized, as in all algal sequences. The sequence alignment was used for the construction of the phylogenetic tree reported in Figure 1.

The present phylogenetic tree derived from green algae PCS confirms the results of the extensive phylogenetic analysis conducted on PCS sequences of various algal and cyanobacterial taxa. As already described in our previous work [63], the midpoint rooted tree is split into two main branches: one containing sequences characterized by the presence of an asparagine residue (Asn, N; see logo 1 in Figure 1) and the other containing sequences characterized by the presence of a glutamic acid (Glu, E) four amino acids upstream the conserved Cys in the catalytic triad.

The first branch (1 in Figure 1) clusters a few sequences clearly separated into two subgroups corresponding to Prasinophytina and Chlorophytina subphyla of Chlorophyta, the first represented by marine microalgae and the latter by a small group of extremophile Volvocales [63]. These sequences have features previously described for prokaryotic PCS [63,64,65], characterized by the inability to phosphorylate the threonine residue upstream of the catalytic Cys (Figure S1) due to the wrong context for the activity of casein kinase 2, and thus by the failure to form the second substrate pocket. These PCS sequences, thoroughly described in *Chlamydomonas eustigma* [66], *Chlamydomonas acidophila* and *Dunaliella salina*, have been hypothesized to have a prokaryotic origin and to derive from horizontal gene transfer [53,54]. These PCS isoforms, also present in marine red algae and diatoms, are characterized by a low number of Cys residues and are also predicted to be more stable than typical land plant PCSs, which may confer an advantage to algae living in extreme environments [63].

The second, larger, branch is in turn divided into two main sub-branches, one (2 in Figure 1) appearing more ancestral and containing only sequences from Volocales, and the another (3 in Figure 1) which groups sequences with characteristics shared by different algal classes (Chlorophyceae, Chloropicophyceae, Pedinophyceae, Pyramimonadophyceae and Trebouxiophyceae) and described also for land plant PCSs. The sequences of both these sub-branches are indeed characterized by the presence of a glutamic acid four amino acids upstream of the catalytic Cys and a cysteine-rich long variable C-terminal domain. In the sequences more widely diffused in all the algal classes, this E residue is preceded by an aspartic acid (Asp, D; see logo 3 in Figure 1), while in the sequences of the branch exclusive to Volvocales this residue is substituted by a glutamic acid (see logo 2 in Figure 1). In this latter restricted group, the catalytic Cys residue is preceded by a glutamine (Glu, Q) while in the majority of the remaining sequences the corresponding position is occupied by an alanine (Ala, A) (Figure S1).

The phylogenetic tree is quite representative of the division within classes and orders: Ulvophyceae are placed on an independent branch, Pyramimonadophyceae, Pedinophyceae, Chloropycophyceae are close to Trebouxiophyceae, and Chlorophyceae cluster on an independent branch split into Volvocales and Sphaeropleales. Two different PCS proteins are apparently present in Prasynophitina (at least as far as Pyramimonadophyceae are concerned) and, among Chlorophytina, three kinds of PCS are present in Chlamydomonadales and only one in the classes Ulvophyceae (few sequences, however, were available for this taxon) and Trebouxiophyceae (in which differences among PCS sequences appear to be due to an independent evolution within orders) as in the order Sphaeropleales.

SaPCS clusters with Scenedesmaceae PCS sequences, very close to the sequence retrieved in the genome of *Tetradesmus obliquus*. In all the Sphaeropleales genomes retrieved in GenBank (accessed on 22 September 2025), only one PCS gene is reported, with the exception of *T. obliquus* (now edited in *Scenedesmus obliquus* UTEX 3031), in which two very close sequences (WIA42506.1 and WIA22074.1) are reported, seemingly due to the presence of two different alleles in this alga indicated as diploid strain [67].

As shown in this analysis and in our former work [63], many algal groups possess more than one PCS gene, as also occurs in land plants [68,69,70], suggesting multiple physiological roles for these enzymes.

The analyses of the Scenedesmaceae annotated genomes available in Phycocosm (https://phycocosm.jgi.doe.gov/phycocosm/home, accessed on 24 September 2025), however, confirm that this algal family harbors a unique gene encoding PCS. The corresponding transcripts, often derived from bioinformatic predictions not yet experimental validated are composed of a variable number of exons ranging from 5 to 9. However, the use of predictive approaches gave rise to alternative interpretations of the putative initial methionine, and consequently to variations in the predicted protein length (Table S2).

### 2.2. Expression Pattern Analysis of SaPCS in wt and Cr-t Strains

**Figure 2:**
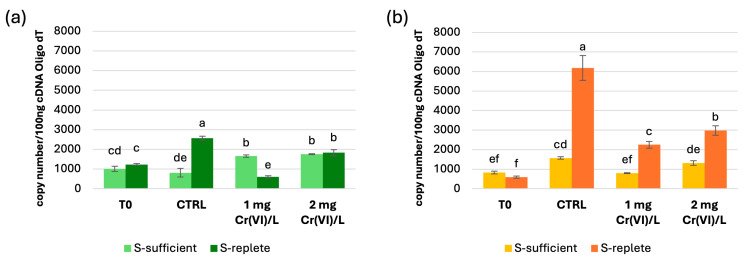
Real-Time qPCR analysis of *SaPCS* expression in the wild-type (**a**) and Cr-tolerant (**b**) strains under S-sufficient and S-replete conditions. The results were reported as the mean value (±standard deviation) of three biological replicates. Statistical analyses were performed using one-way ANOVA followed by Duncan’s post hoc test (*p* < 0.05). Different letters denote a significant difference between treatments within a strain.

Absolute quantification using Real-Time qPCR analysis of *SaPCS* in wt and Cr-t strains showed significant variations in transcript levels in the different conditions tested. No differences in gene expression were observed at the end of the pre-cultures (T0) in wt e Cr-t strains under both S-replete and S-sufficient conditions (Figure 2), indicating that S-starvation per se does not affect *SaPCS* transcription. On the other hand, in S-sufficient condition *SaPCS* is significantly up-regulated by Cr(VI) exposure in the wt strain, whereas in the Cr-t strain *SaPCS* transcription is mainly enhanced in response to nutrient resupply rather than Cr(VI) exposure (Figure 2a,b). Under S-replete conditions, both strains showed enhanced *SaPCS* transcription mainly after medium renewal (control, CTRL in Figure 2a,b), although to different extents (1221 ± 55 vs. 2562 ± 100 copies in the wt and 594 ± 56 vs. 6174 ± 635 copies in the Cr-t strain, in T0 vs. CTRL), rather than in response to Cr(VI) exposure, which caused a minor upregulation compared to the controls. Interestingly, an increase in PC synthesis was observed after transfer to fresh medium containing Zn and Cu, as reported by Grill and coworkers (1988) [71], who suggested a role for PCS in metal homeostasis. Although it is known that the *PCS* gene is constitutively expressed and the regulation of PCs synthesis mainly occurs at protein activity level, weak modulation of gene expression has been reported following metal exposure both in Arabidopsis and rice [34,72].

This complex transcriptional pattern observed in both strains across different conditions assayed suggests that SaPCS might not be directly involved in Cr(VI) detoxification, but rather it may play a role in S metabolism and cellular homeostasis during stress.

### 2.3. Western Blot Analysis of SaPCS

**Figure 3:**
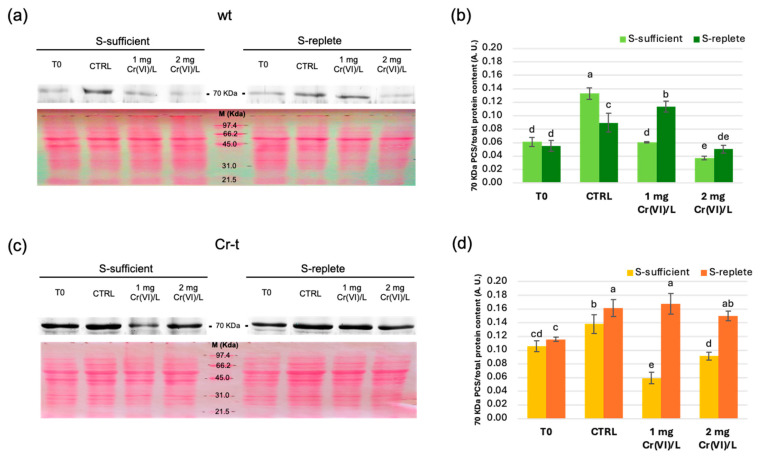
Western blot analysis (**a**,**c**) and relative quantification (**b**,**d**) of full-length PCS (70 kDa) in the wild-type (**a**,**b**) and Cr-tolerant (**c**,**d**) strains. The results were reported as the mean value (±standard deviation) of three independent experiments. Proteins extracted from both S-sufficient and S-replete cells for each strain were processed in the same gel. For each strain, the upper image shows SaPCS immunodetection, while the lower image shows Ponceau staining, in which the central line corresponds to the molecular weight marker. Statistical analyses were performed using one-way ANOVA followed by Duncan’s post hoc test (*p* < 0.05). Different letters denote a significant difference between treatments within strain.

**Figure 4:**
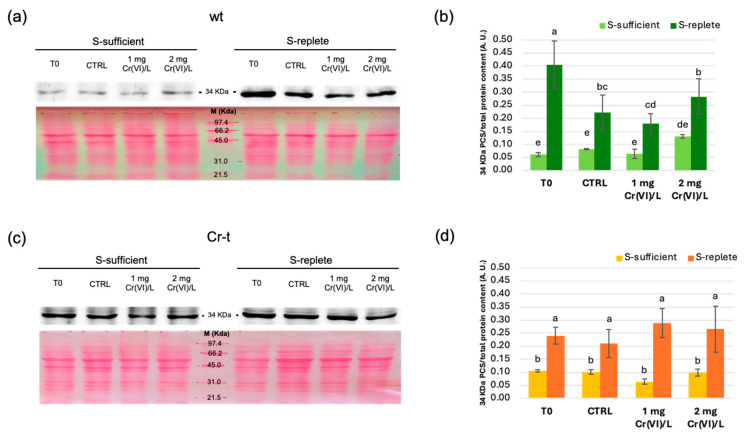
Western blot analysis (**a**,**c**) and relative quantification (**b**,**d**) of PCS (34 kDa) in the wild-type (**a**,**b**) and Cr-tolerant (**c**,**d**) strains. The results were reported as the mean value (±standard deviation) of three independent experiments. Proteins extracted from both S-sufficient and S-replete cells for each strain were processed in the same gel. For each strain, the upper image shows SaPCS immunodetection, while the lower image shows Ponceau staining, in which the central line corresponds to the molecular weight marker. Statistical analyses were performed using one-way ANOVA followed by Duncan’s post hoc test (*p* < 0.05). Different letters denote a significant difference between treatments within strain.

A Western blot analysis was performed using a polyclonal antibody raised against *A. thaliana* PCS1 (AtPCS1, 56 kDa) [73] to evaluate SaPCS protein expression in wt and Cr-t strains under S-sufficient and S-replete conditions. The analysis unexpectedly revealed immunoreaction signals corresponding to two proteins, one with an apparent molecular weight of approximately 70 kDa (Figure 3), consistent with the molecular weight of the deduced full-length SaPCS protein as predicted by ProtParam [74], and another with an apparent molecular weight of nearly 34 kDa (Figure 4).

Immunoblot analysis showed that the full-length SaPCS protein (70 kDa) is highly expressed in the Cr-t strain (Figure 3). Its expression increased in both strains upon nutrient resupply, and no further increase was observed in the presence of Cr(VI) under both S-sufficient and S-replete conditions. Under the S-sufficient condition, a decrease in protein abundance was observed in the Cr-t strain exposed to 1 mg Cr(VI)/L and in the wt strain treated with 2 mg Cr(VI)/L. Under the S-replete condition, a significant increase in SaPCS expression was detected in the wt treated with 1 mg Cr(VI)/L, in agreement with the RT-qPCR data (Figure 2). These results indicate that the SaPCS protein expression is primarily driven by nutrient availability rather than by Cr(VI) exposure.

The abundance of the putative smaller isoform of SaPCS (34 kDa) remained relatively stable under S-sufficient conditions, with apparently higher basal levels in the Cr-t strain. Higher abundance was observed in both strains under S-replete conditions, with a more pronounced increase in the wt compared to the Cr-t strain (8-fold vs. 2.5-fold relative to the corresponding S-sufficient conditions, respectively). After 24 h from nutrient re-supply, levels of this “short” protein significantly decreased in wt S-replete cells (both for control and Cr(VI)-treated cells), while remaining stable in the Cr-t strain. In both strains, however, protein levels significantly higher in S-replete than in the corresponding S-sufficient cells.

To summarize, both protein isoforms recognized by the AtPCS1 antibody are expressed at a higher basal level in the Cr-t strain and are more affected by S-availability than by Cr(VI) exposure. Strain-specific differences were mainly observed for the 34 kDa protein isoform, which was expressed at lower basal levels in the wt under S-sufficient conditions but reached levels comparable to the Cr-t strain following S-starvation.

Since SaPCS and AtPCS1 share high similarity in the highly conserved N-terminal domain (containing the catalytic triad) but strongly diverge in the variable C-terminal domain (Figure S2), it is reasonable to assume that the smaller immunostained band corresponds to a PCS protein containing only the conserved catalytic domain and lacking the variable C-terminal domain.

“Half-size” PCS proteins were originally described in cyanobacterial PCSs [42,53,64,65,75]. These proteins differ from land plants PCS N-terminal domains in some features affecting the post-transcriptional regulation and the formation of the second substrate pocket [39], making them unable to synthesize long chain PCs. They were thus suggested to be involved in GSH metabolism rather than in PCs synthesis [39]. Nowadays, since more genomic data have become available, in silico analyses have revealed that PCS from the cyanobacterium *Nostoc* sp. (NsPCS), previously considered a model for prokaryotic enzymes, is not the only PCS in cyanobacteria, which also possesses a protein sharing features with the eukaryotic enzyme [63]. Moreover, as stated above, some algal groups possess two or three PCS genes in their genomes, and certain marine and extremophile green algae (branch 1 in the phylogenetic tree, Figure 1) additionally encode smaller PCS-like protein isoforms [63]. However, this is not the case for *Scenedesmus*. In the genome of *Scenedesmus obliquus* (synonym of *S. acutus*) UTEX393 (https://phycocosm.jgi.doe.gov/Sceobl393_2/Sceobl393_2.home.html, accessed on 10 June 2025) and in other Sphaeropleales genomes available in public databases, only a single *PCS* gene has been identified, encoding a 70 kDa protein.

### 2.4. Analysis of SaPCS Splicing Variants

Based on the above observations, we investigated the biosynthetic mechanism and potential function of the 34 kDa protein isoform. Although a second *PCS* gene is absent in Sphaeropleales genomes, a shorter protein could be produced either through a proteolytic cleavage or alternative mRNA splicing. Both mechanisms are plausible, since protein rearrangement has been reported during S-starvation [76,77], and alternative splicing of the PCS transcript has been documented in land plants as *S. rostrata* [52] and *O. sativa* [34], as well as in extremophile green algae *C. acidophila* and *D. acidophila* [53].

Regarding possible protein fragmentation, it is known that AtPCS1 protein from *A. thaliana* (56 kDa) contains instability points at Glu283 and Asp372, which upon limited proteolysis analysis generate 34 and 44 kDa protein isoforms, respectively. Both isoforms retain the N-terminal domain, which is necessary and sufficient for catalytic activity, while losing the evolutionarily divergent C-terminal domain, demonstrated to be involved in the responsiveness to a wide range of heavy metals [23,73,78]. This was further confirmed through C-terminal truncation experiments, which identified the region responsible for Zn-dependent PC formation in the AtPCS1 C-terminal domain [79]. Additionally, As-specific activation of PC synthesis was shown to occur in a small region of the C-terminal domain [80].

Another possible hypothesis for the formation of the smaller isoform lies in the possibility of alternative mRNA splicing, a crucial mechanism in eukaryotic gene expression that allows a single gene to produce multiple protein isoforms with distinct functional properties [81]. The detection of a weak band, shorter than expected (441 bp), in an endpoint PCR, performed using primers annealing to the 5′ region using cDNAs obtained from S-replete samples exposed to 1 mg/L Cr(VI) (Figure S3) prompted further investigation into potential mRNA rearrangements.

**Figure 5:**
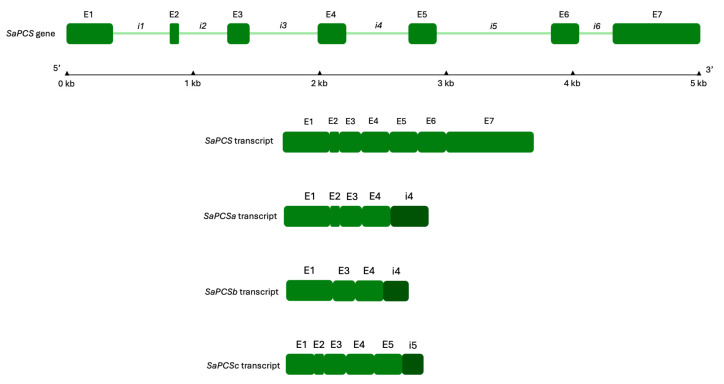
Structures of *SaPCS* genomic sequence and alternatively spliced transcript variants. The *SaPCS* genomic sequence (GenBank accession N.: MF278027) and the transcriptional isoforms *SaPCSa*, *SaPCSb* and *SaPCSc* (GenBank accession N.: PP974219, PP974220 and PQ276120, respectively) were used to model the gene and transcript structures. Green boxes represent exons, numbered E1–E7, whereas lines represent introns (i1–i6). Exons and introns lengths are given in Kb and are drawn to scale. Dark green boxes represent retained introns.

Since the 34 kDa protein isoform presumably corresponds to the conserved N-terminal domain encoded by exons 1–4, we designed two reverse primers annealing to intron 4 and intron 5 to investigate the presence of putative alternative transcripts (Table 1 and Figure 5). All these reverse primers were first validated on *S. acutus* genomic DNA in PCR reactions using the forward primer SaPCS Fmet, which anneals to the region containing the putative initial methionine codon of the full-length isoform (exon 1), yielding amplicons of the expected sizes. When applied to cDNA, SaPCS Fmet primer paired with both reverse primers annealing to intron 4 (SaPCS SplR1 and SaPCS SplR2) produced amplicons of 951 bp and 1235 bp, respectively. In contrast, the reverse primer annealing to intron 5 (SaPCS SplR3) did not amplify with SaPCS Fmet primer but produced an amplicon when paired with the forward primer SaPCS F1, which anneals to exon 1, 80 nucleotides downstream of the initial methionine codon.

The 951 bp amplicon obtained with SaPCS Fmet-SaPCS SplR1 primer pairs was found to be part of the same transcript obtained with the downstream primer SaPCS SplR2 (1235 bp); this transcript was designated *SaPCSa* (GenBank accession N.: PP974219). Interestingly, in samples from both strains exposed to 1 mg/L Cr(VI) for 24 h under S-replete conditions, the same primer pair produced a second amplicon that was 73 bp shorter and lacked exon 2. This transcript, designed *SaPCSb* (GenBank accession N.: PP974220), was shown to correspond to the sequence of the shorter band observed in endpoint PCR in both wt and Cr-t strains under S-replete conditions following 24 h of Cr(VI) exposure (see above and Figure S3). The amplicon (1215 bp) obtained using SaPCS F1 (exon 1) and SaPCS SplR3 (intron 5) primers was designed *SaPCSc* (GenBank accession N.: PQ276120) and comprised the first four exons and part of intron 5.

The structure of the SaPCS transcripts generated by putative alternative splicing are shown in Figure 5. For each transcript, the observed size corresponded to that expected in the case of 5′ intron retention.

The *SaPCSa* transcript contains two putative premature termination codons (PTCs) at the position of 1005 bp and of 1077 bp (51 bp and 123 bp downstream of the SaPCS SplR1 primer site, respectively). This PTC-containing *SaPCSa* transcript generated by retention of the fourth intron of the *SaPCS* gene leads to the premature termination of the CDS (Figure 5), suggesting the synthesis of an unusually smaller protein isoform. Despite this truncation, SaPCSa retains the N-terminal PCS domain (Pfam accession number: PF05023), including the conserved C–H–D catalytic triad. The putative SaPCSa polypeptide, comprising 334 amino acids when translated up to the first stop codon, has a calculated molecular weight of 36.56 KDa.

The alternatively spliced *SaPCSc* transcript also contains a PTC at the position 1137 bp due to the retention of the fifth intron of the *SaPCS* gene, leading to the premature termination of the C-terminal domain of the protein, even maintaining the N-terminal domain, similarly to *SaPCSa*. However, this transcript appears to lack the initial part of the N-terminal domain, as no amplification from cDNA was obtained using primers annealing to the region containing the initial methionine. Consequently, it remains unclear whether translation starts from a methionine codon located upstream or downstream of the canonical SaPCS start codon, or whether a non-AUG translation initiation site (TIS) is utilized. The use of non-AUG TISs is a widespread phenomenon in both prokaryotes and eukaryotes and represents a mechanism to obtain multiple transcripts/protein isoforms from a single gene, especially in processes involved in development and stress response [82]. For these reasons, it is difficult to reliably predict the hypothetical weight of the putative translated SaPCSc protein isoform.

Finally, the alternatively spliced *SaPCSb* transcript lacks the 73 bp exon 2 and retains the intron 4. Consequently, a frameshift occurs in this transcript, leading to PTC formation. Thus, the resulting *SaPCSb* polypeptide lacks not only the Cys-rich region, but also the Cys, His and Asp residues of the characteristic catalytic triad at the N-terminal PCS domain (Figure 5), unless its translation starts from a non-AUG TIS on frame +3, or proceeds via a readthrough event [83]. In these latter cases, the complete catalytic domain could indeed be restored, giving rise to putative protein isoforms ranging between about 29 and 32 KDa. Putative translation of these truncated variants is reported in Figure S4.

Interestingly, all the alternative transcripts maintained both the threonine residue (codified by the first codon on exon 3) located upstream of the catalytic Cys and the arginine residue located downstream of the catalytic aspartic acid (encoded by the third codon of exon 5 or, alternatively, of intron 4; Figure S4). These residues have been reported to be essential for the formation of the second substrate pocket [39].

The phenomenon of alternative splicing in the PCS *genes* has been studied in different plant crops, such as the legume *S. rostrata* [52] and *O. sativa* [34,68], particularly under heavy metal stress conditions. The single copy of the *SrPCS* gene in *S. rostrata* produces four alternatively spliced transcripts. Among these, the SrPCS1 and SrPCS3 proteins contain the complete N-terminal domains, including the C-H-D catalytic triad. In contrast, the SrPCS2 and SrPCS4 proteins have only the Cys residue of the catalytic triad, indicating that specific splicing variants play distinct roles in heavy metal detoxification [52]. In rice, alternative splicing variants have been isolated in two independent studies on *O. sativa indica* [34] and *japonica* [68] cultivars. Some of the variants isolated by Das et al. (2017) [34] were initially described as a shorter *OsPCS2* isoform but were further identified by Uraguchi and colleagues (2017) [68] as *OsPCS1* splicing variants. A shorter *OsPCS2* isoform, lacking the C-terminal domain, was also identified with no alternative transcript [68]. The alternatively spliced transcripts described for rice contain PTCs, due to intron retention, which alters the reading frame and leads to early termination of the coding sequence. The resulting polypeptides lack the N-terminal domain containing the C-H-D catalytic triad or the C-terminal domain, which is involved in determining metal-specific sensitivity [34,68]. Das et al. (2017) [34] postulated that the unproductively spliced mRNAs might compete functionally with their full-length mRNA counterparts, contributing to a post-transcriptional regulatory mechanism of protein expression [34]. Furthermore, the researchers proposed that in rice, tissue-specific expression of splicing variants under Cd stress controls the levels of PTC-containing unproductive transcripts in complementation to the expression of the full transcript [34]. The analysis of the two rice isoforms (*OsPCS1full* and *OsPCS2*), as well as the alternative splicing variants of *OsPCS1* (*OsPCS1a*, *OsPCS1b*, *OsPCS1c*), in complementation experiments in both yeast and plants indicated that only *OsPCS1full* possesses PCS activity in response to Cd and As. In contrast, *OsPCS2* showed only a low Cd-dependent PCS activity, while the truncated *OsPCS1* variants showed no significant PCS activity [68]. The authors postulated that these different variants might also play a role in metal distribution and/or reallocation within the plant.

Alternative polyadenylation in UTR with Open Reading Frame shifting, originating different PCS transcripts, was also shown to occur in the extremophile green algae *C. acidophila* and *D. acidophila* [53].

These alternative transcription mechanisms have also been reported to influence protein subcellular localization both in animals and plants [83,84,85,86,87,88]. Interestingly, in the *S. acutus SaPCSb* variant the possible shifting from frame +1 to frame +3 due to the exon 2 skipping generated the sequence “AARAAAAAA” (highlighted in gray in Figure S4), which closely resemble the predicted sequence around the cleavage site for mitochondrial transit peptide in *Chlamydomonas reinhardtii* [89].

The functional implications of these truncated variants could be significant, since many mRNA translation modulating strategies have recently been discovered that subvert the dogma “one gene, one mRNA one protein” and demonstrate that context cues can heavily affect translation [82,83,87,88,90]. This translation plasticity seems to especially influence proteins involved in stress response. Alternative splicing or use of non-AUG TISs and readthrough events can indeed generate protein isoforms with distinct properties, potentially allowing the organism to fine-tune its response to varying heavy metal stresses [83,84,85,86,87,88].

Similarly to what has been hypothesized for land plants, the truncated form of *SaPCS* transcripts, *SaPCSa-c* in *S. acutus*, could play a role in the regulation of full-length *SaPCS.* Moreover, the wide range of post-translational regulations which can intervene on its charge could explain the modulation of this enzyme constitutively expressed throughout all the kingdoms of life, even when it is not apparently required for metal detoxification, and whose transcription is only subject to little variation.

Even the role of canonically transcribed isoforms is not fully understood in plants. In *A. thaliana* AtPCS2 is constitutively expressed in all tissue, albeit at a lower level than AtPCS1, and in experiments of complementation in yeast it has been proven to contribute to Cu toxicity, notwithstanding the failure in producing PCs [43]. The short OsPCS2 lacking the C-terminal domain produced PCs in response to Cd in complementation experiments conducted in yeast, but not in plants, albeit conferring partial tolerance to Cd. Among the few studies in this regard conducted in microalgae, Diaz and colleagues (2022) [54] demonstrated that CaPCS2 from *C. acidophila* is mostly linked to the detoxification of various stresses involved in ROS production, rather than to metal sequestration/accumulation. CaPCS2 belongs to the half-PCS found in the extremophile green algae sequences (branch 1 in the phylogenetic tree in Figure 1), which has been hypothesized to derive from prokaryotes via horizontal gene transfer [53,54]. The prokaryote half-PCS is not able to form the second substrate pocket and is rather involved in GSH metabolism [39], the principal actor in counteracting ROS damage. These isoforms, initially described as prokaryotic PCS, but actually demonstrated to be present in different eukaryotic algal taxa [53,54,63,66], play a role in response to multiple abiotic stresses through GSH turnover, rather than in metal chelation through the synthesis of PCs. They have likely been maintained in extremophile organisms living in environments where they are constantly forced to deal with ROS-producing stresses.

It is thus not excluded that, although only one gene is found in *S. acutus*, shorter isoforms with distinct physiological roles could be routinely generated through alternative transcriptional processes. Similarly to what has been reported for AtPCS2, the short 34 KDa SaPCS isoform is likely associated with tolerance, since its expression under the S-sufficient condition is higher in the Cr-t strain than in wt. In the latter, it became more expressed in S-replete cells, a condition in which this strain transiently increases its tolerance. In this regard, it is important to note that S-starvation causes oxidative stress due to the deterioration of the Fe/S clusters of cytochromes and the consequent alteration of the electron transport chain, both in chloroplast and in mitochondria [91,92], and this may explain why the short SaPCS isoform level increased in response to S nutritional stress.

Unfortunately, due to the high degree of sequence similarity between the mRNAs of *SaPCSa*, *SaPCSb* and *SaPCSc*, the identification of specific primers capable of distinguishing the expression by RT-qPCR of isoforms due to alternative splicing has proven to be unfeasible. For this reason, it was not possible to correlate a specific short transcript to the short protein isoform.

### 2.5. Mass Spectrometry Analyses of PC Production

The lack of PC induction following Cr(VI) exposure was already reported in *Scenedesmus acutus* [93], but was never investigated in S-replete cells upon S-starvation.

To evaluate if PCs production could have a role in the transient increase in chromium tolerance observed in *S. acutus* after S-starvation [60,61,62], PC content was quantified by mass spectrometry, in both strains, at the end of 72 h pre-culture in +S and −S medium and after 24 h Cr(VI) exposure at the respective LOEC in S-sufficient and S-replete conditions. Notably, in Cr-t cells grown in S-replete conditions, a great increase in Cys production was observed, without any effect on negative feedback regulation of the S uptake and assimilation pathway [62]. These data prompted us to hypothesize that PCs could not only play a direct role in metal detoxification but could also act as a reduced S storage form, allowing for the accumulation of the elevated Cys levels produced after nutrient resupply [60,62]. This hypothesis was corroborated by the observed increase in the “short” protein isoform, containing the catalytic domain but presumably lacking the C-terminal domain involved in metal recognition.

In addition, PCs were also quantified in wt cells pre-cultivated for 72 h in standard medium and subsequently exposed to 2.25 µM Cd (provided as Cd(NO_3_)_2_) for 24 h, serving as a positive control [93].

**Figure 6:**
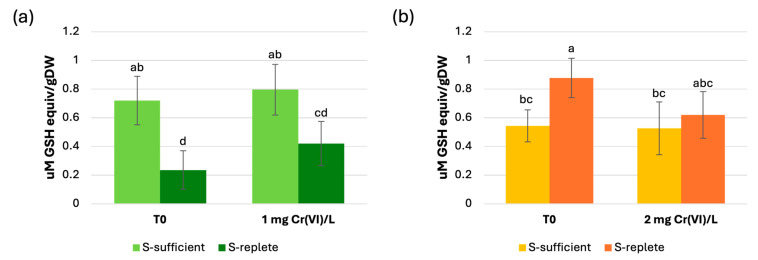
Quantification of PC_2_ in the wild-type (**a**) and Cr-tolerant (**b**) strains after pre-culture and 24 h exposure to Cr(VI) (1 and 2 mg/L) under S-sufficient or S-replete conditions. The results were reported as the mean value (±standard deviation) of three biological replicates. Statistical analyses were performed cumulatively on the two strains using one-way ANOVA followed by Duncan’s post hoc test (*p* < 0.05). Different letters denote a significant difference between treatments and strains.

Our analysis revealed only a weak production of PC_2_: under S-sufficient conditions, both strains exhibited basal PC_2_ levels that were not significantly different (0.72 ± 0.17 and 0.54 ± 0.11 µM GSH equiv/gDW respectively for the wt and Cr-t strains). These levels did not increase significantly after Cr(VI) exposure (Figure 6a). At T0 under S-replete conditions, wt cells showed a marked reduction in PC levels (0.23 ± 0.13 µM GSH equiv/gDW), whereas, unexpectedly, Cr-t cells showed a slight but significant increase (0.88 ± 0.13 µM GSH equiv/gDW). In both nutritional conditions, no significant induction of PC_2_ production was observed upon Cr(VI) exposure (Figure 6a).

Nevertheless, although statistically significant, the differences in PC levels observed in response to Cr(VI)-treatments were negligible when compared with the strong PC accumulation in Cd-treated wt cells. In this sample, PC content reached 11.15 ± 2.9 µM GSH equiv/gDW (Figure S5), results similar to those reported by Torricelli et al. (2004) [93], highlighting the strong induction of PC biosynthesis by Cd in contrast to Cr(VI).

Given these observations, we can considerably exclude that in *S. acutus* PCs are involved in Cr(VI) direct detoxification and that they can be utilized as a means to store reduced S when the nutrient is available and Cys is produced in excess.

Although not observed in *S. acutus* [59], the induction of PC production by Cr treatment is frequently reported as a species- or organ-specific response. Indeed, several studies have shown that Cr exposure can induce PC production [33,34,35,36,37], whereas in some model plants, no modulation, or even a reduction, in PC levels has been reported [94,95]. For instance, in *O. sativa* roots, PC levels in Cr-stressed plants were similar to those of untreated plants [94]. Similarly, in the roots and shoots of *Nicotiana tabacum*, Cr exposure did not induce the production of GSH or PCs [95].

On the other hand, GSH plays a central role in plant cellular detoxification of xenobiotics and heavy metals through the activation and conjugation of toxic compounds [96,97]. This process is catalyzed by glutathione S-transferases [98,99] and the resulting conjugates are subsequently sequestered into the vacuole, thereby mitigating cytotoxicity [5,6].

Through thiol-based chelation, GSH leads to Cr–GSH complexes [100], reducing the pool of free cellular chromium [101,102]. In addition, its antioxidant capacity counteracts Cr-induced ROS via the ascorbate–GSH cycle, thereby preserving cellular homeostasis [103,104,105].

Considering these observations and previous findings on GSH dynamics in response to Cr(VI) in *S. acutus* [62], it is possible to hypothesize that GSH, rather than PCs, is directly involved in the detoxification of Cr(VI) in this alga.

Indeed, under S-sufficient conditions, both the wt and the Cr-t strains showed similar GSH levels at the end of the pre-culture (T0). During recovery in the absence of Cr(VI) treatment, GSH levels increased significantly only in the Cr-t strain, whereas Cr(VI) exposure induced a significant increase in GSH in both strains [62], consistent with the dual role of GSH in metal chelation and ROS scavenger via the ascorbate–GSH cycle.

Under S-replete conditions, both strains initially showed low GSH levels but later exhibited a marked increase following the S resupply. Cr(VI) had no additional effect under these conditions, indicating maximal activation of GSH biosynthesis [62]. Moreover, GSH production efficiency (GSH/S ratio) was similar in both strains and unaffected by S availability during pre-culture, but increased significantly after Cr(VI) exposure, except in wt under S-replete conditions, where no variation was observed [62].

In this scenario, and in the light of recent findings on CaPCS2 from *C. acidophila* [54] the 34 kDa PCS derived from some of the shorter transcripts identified in the present manuscript could assume the role analogous to the “half-PCS protein” described in some algal genomes (branch 1, Figure 1), which are supposed to play a role in counteracting multiple ROS-generating stresses, not through PC production.

Overall, in the wt strain, GSH appears to function as a reactive, on-demand resource that is more susceptible to depletion under prolonged Cr(VI) stress, whereas in the Cr-t strain it acts as a readily available reserve, anticipating exposure and enhancing Cr(VI) sequestration while maintaining redox homeostasis.

## 3. Materials and Methods

### 3.1. In Vitro Culture, Sulfur Starvation and Chromium Treatments of Scenedesmus acutus

Two strains of the freshwater green alga *S. acutus* (M.) with different hexavalent chromium (Cr(VI)) sensitivity, namely wild-type (wt) and chromium-tolerant (Cr-t), were used as experimental material. The Cr-t strain was isolated by treating the wt population with a sublethal concentration (1 mg Cr(VI)/L) for 3 months [57].

Synchronized axenic cultures of both strains of *S. acutus* were grown in US EPA (1978) liquid culture medium at pH = 7.2 ± 0.1, with both micro and macronutrients at double the concentration indicated [61,106,107]. No organic matter was present in the medium at the beginning of the culture.

**Figure 7:**
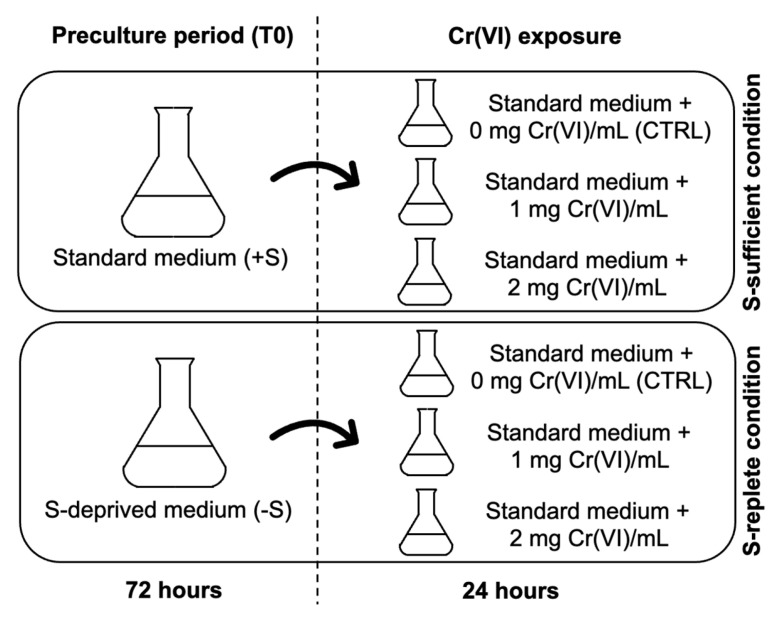
Schematic representation of the experimental setup used in this work.

The S-starvation and Cr(VI) stress treatments were performed as reported in Ferrari et al. (2022) [15]. Briefly, cells were precultured for 72 h (T0) in standard (+S) and sulfate-deprived (−S) media, in which MgSO_4_ was omitted and magnesium restored with MgCl_2_. After 72 h, pre-culture in +S (un-starved cells) or in −S medium (starved cells) of both strains were collected by centrifugation, washed and resuspended at 3 × 10^6^ cells/mL [57,60,93] in fresh standard medium and exposed for 24 h to 0 (control, CTRL), 1 and 2 mg Cr(VI)/L, supplied as K_2_Cr_2_O_7_. From now on, “S-sufficient cells” will indicate un-starved cells which were transferred to and maintained in +S medium, while “S-replete cells” will indicate starved cells which were transferred to +S medium. Analogous meaning will be attributed to the terms “S-sufficient conditions” and “S-replete conditions” when speaking of experimental conditions (Figure 7). The selected Cr(VI) concentrations correspond to the LOEC inhibiting growth for the wt and Cr-t strains, as previously determined [59]. All treatments and controls were performed in triplicate. Cultures were maintained in a climate-controlled chamber (23 ± 1 °C) under a 16:8 h light:dark cycle, with an irradiance of 230 µmol m^−2^ s^−1^ and continuous aeration with sterile-filtered air.

### 3.2. Total RNA Extraction and cDNA Synthesis

Culture aliquots from various treatments of both strains were collected by centrifugation, twice washed with double distilled water, frozen in liquid nitrogen, lyophilized, mortar ground in liquid nitrogen and stored at −80 °C before RNA extraction. Total RNA was extracted as described by Ferrari et al. (2022) [15]. RNA was quantified by Nanodrop (ND-1000 UV-VIS spectrophotometer; NanoDrop Technologies, Wilmington, DE, USA), and integrity was checked by an Agilent 2100 Bioanalyzer (Agilent Technologies, Santa Clara, CA, USA). Only RNA samples with an RNA integrity number ≥ 7 were used for cDNA synthesis. The cDNA synthesis was performed by SuperScript™ III using oligo-dT primers (Invitrogen, Carlsbad, CA, USA) from 1 µg of total RNA, according to the manufacturer’s instructions.

### 3.3. Phylogenetic Analysis

Evolutionary history of green algae PCS was inferred as described in Ferrari et al. (2024) [63]. The Maximum Likelihood method was used, along with the JTT matrix-based model [108], and analysis was conducted using MEGA 11 software [109]. The initial tree for the heuristic search was obtained automatically with Neighbor-Join and BioNJ algorithms applied to a matrix of pairwise distances estimated by using the JTT model. The topology with the superior log likelihood value was selected. The validity of the obtained phylogenetic tree was checked through the bootstrap test using 1000 replicates. The alignments were performed by using ClustalW [110], and tree representation was modified through iTOL “interactive Tree Of Life” [111]. The sequence of SaPCS was used as a query against the NCBI database (www.ncbi.nlm.nih.gov/BLAST, accessed on 24 September 2025) for proteomic accession through BlastP analysis [112]. Searches were performed across the different green algal taxa present in the database. The GenBank accessions of the sequences used for the analysis are reported in Table S1. The consensus patterns in these candidate sequences were checked with PROSITE (https://prosite.expasy.org/scanprosite/, accessed on 30 September 2025) to include only sequences of true PCSs, given that many analyzed genomes have not been completely annotated.

### 3.4. Sequence Analyses

Retrieved sequences were aligned by ClustalX [110] and MEGA 11 software [109] and visualized through Genedoc [113]. Sequence homology analyses were performed by using Blastp (www.ncbi.nlm.nih.gov/BLAST, accessed on 24 September 2025), ClustalX [110] and WebLogo [114,115].

The hypothetical molecular weights of the deduced SaPCS proteins were analyzed with ProtParam tool [74].

### 3.5. Absolute Quantification Using Real-Time qPCR

Expression of *SaPCS* was evaluated by absolute quantitative Real-Time PCR analysis (aqPCR). The forward *SaPCS* qPCR F (5′-GTCAGCATTGTGGAAGGGGA-3′) and reverse *SaPCS* qPCR R (5′-CACCGTCAGACAAACCTCCA-3′) primers were designed on exon 7 using Primer Express™ Software v3.0.1 (Applied Biosystems, Foster City, CA, USA) and selected according to their robustness, specificity and consistency. The efficiency of the primers for the *SaPCS* (GenBank: MF278027.2) gene was 99%. The copy number of *SaPCS* was evaluated using a serially diluted standard of known concentration (ranging from 10^9^ to 10^1^ copies per μL, with a 10-fold difference) to generate a calibration curve with a linear relationship between Ct and the logarithm of the initial amount of total template DNA [116]. The dsDNA standard was obtained by PCR [117] with the same primers used for subsequent quantification. The initial copy number for generating the calibration curve was calculated according to Whelan et al. (2003) [118].

Amplification reactions were performed using the Select SYBR^®^ Green PCR Master Mix (Applied Biosystems, Foster City, CA, USA) in a STEP ONE instrument (Applied Biosystems, Foster City, CA, USA). The target gene copy number was determined by reading the standard series with the Ct values of each sample and reported as the number of molecules per nanogram cDNA. Statistical analysis was performed using one-way ANOVA with the Duncan post hoc test (*p* < 0.05) after the Shapiro–Wilk normality test and Levene’s homoscedasticity test using SPSS 25 software (IBM Corporation, Armonk, NY, USA).

### 3.6. Western Blots

Total proteins were extracted from cells collected through centrifugation and washed three times in double distilled water. The obtained pellets were resuspended in 3 mL 50 mM TRIS-HCl pH 7.5 and disrupted using “One Shot” Cell Disrupter (Constant Systems Ltd., Daventry, UK) at 2700 bars, one time. After rupture, homogenates were brought to a final volume of 5 mL and supplemented with both dithiothreitol (DTT) and phenylmethylsulphonyl fluoride (PMSF) to reach the final concentration of 1 mM. Proteins were ammonium sulfate-precipitated (by adding 436 mg ammonium sulfate to 1 mL extract) and final pellets were resuspended in 200 µL Laemmli sample buffer (LSB) [119]. Protein concentration was determined according to Bradford (1976) [120], using bovine serum albumin as a reference. Aliquots of protein extracts (~25 μg of total protein per well) were separated on a 12% SDS–polyacrylamide gel [119]; broad range SDS PAGE Standards (Bio-Rad Laboratories, CA, USA) were used as reference for MW determination. After running, proteins were electro transferred at 400 mA V for 2 h to a Zeta-Probe blotting membrane (Bio-Rad Laboratories, Inc., Hercules, CA, USA), using a Mini Trans-Blot cell apparatus (Bio-Rad Laboratories, Hercules, CA, USA) in a cold room. Protein loading and transfer efficiency of extracts were checked by Ponceau-S staining and membrane images were acquired with a Kodak DC40 camera (Kodak, Rochester, NY, USA). After membrane washing, Western blot analyses were performed with a polyclonal antibody (diluted 1:10,000 in blocking buffer and probed for 2 h at room temperature) raised against *A. thaliana* PCS1. As references for the immunoblotting specificity/detection, recombinant *A. thaliana* PCS1 (AtPCS1, 56 kDa) [73], was employed. Immunoreactivity was visualized using a goat anti-rabbit IrDye 680 (LI-COR Biosciences GmbH, Bad Homburg vor der Höhe, Germany) diluted 1:10,000 for 1 h at room temperature working in subdued light). Detection was performed through infrared fluorescence (NIRF) with Odyssey DLx imaging system (LI-COR Biosciences GmbH). The intensity of each target band was normalized to the total protein amount of the corresponding Ponceau-stained lane [121,122]. The final value of the relative expression was expressed as target band intensity/total lane intensity (arbitrary unit, A. U.). Comparisons between treatments were conducted exclusively between samples present on the same gel to avoid intrinsic technical variability between different gels. Three independent biological replicates were performed. Statistical analysis was performed using one-way ANOVA with the Duncan post hoc test (*p* < 0.05) after the Shapiro–Wilk normality test and Levene’s homoscedasticity test using SPSS 25 software (IBM Corporation, Armonk, NY, USA).

### 3.7. Identification of SaPCS Transcripts

PCR analyses were conducted on cDNA samples using primers indicated in Table 1. PCR conditions and sequence analysis were performed according to Ferrari et al. (2020) [107].

### 3.8. Quantification of PC Production by Mass Spectrometry

Mass spectrometry analyses for quantification of PC production were performed on proteins extracted from algae of both strains grown in S-sufficient or S-replete condition, exposed to 1 and 2 mg Cr(VI)/L LOEC respectively for the wt and Cr-t strain. An algal culture of wt strain cells grown in S-sufficient condition and exposed to Cd 2.25 µM (supplied as Cd(NO_3_)_2_) for 24 h [93] was used as a positive reference. For each condition, algal cells from 400 mL culture were collected by centrifugation and washed two times in double distilled water, and the final pellets were resuspended in 800 μL 5% (*w*/*v*) sulfosalicylic acid and disrupted using a “One Shot” Cell Disrupter (Constant Systems Ltd., Daventry, UK) at 2700 bars, one times. The homogenates were centrifuged for 20 min at 12,000× *g* at 4 °C, and the recovered supernatant was rapidly frozen in liquid nitrogen and stored at −80 °C till use for analysis.

The identity of putative PCs was verified using an LC/MSD XT mass analyzer (Agilent, Santa Clara, CA, USA). Liquid chromatographic elution was carried out on an AERIS peptide 3.6 μm XB-C18 100, 150 × 2.1 mm column (Phenomenex, Torrance, CA, USA), using a gradient solvent system [solvent A, aqueous 0.1% (*v*/*v*) formic acid; solvent B, 0.1% (*v*/*v*) formic acid in acetonitrile] as follows: solvent B was set at 0.5% for 12 min, raised with a linear gradient to 10% in 3 min, raised with a linear gradient to 20% in 5 min, and then raised with a linear gradient to 99% in 1 min. Solvent B was maintained at 99% for 3 min before column re-equilibration (10 min). The column temperature was set at 40 °C and the flow rate was 200 mL/min. The injection volume was 10 μL. PCs detection was carried out in Single Ion Monitoring scan mode with an LC/MSD XT mass analyzer (Agilent, Santa Clara, CA, USA) with a gas (nitrogen, 99.999% purity) temperature of 300 °C, gas flow of 10 L/min, and a nebulizer operating at 25 psi. The sheath gas was delivered at a flow rate of 10 L/min with a gas temperature of 360 °C, capillary voltage of 4 kV, and a nozzle voltage of 2 kV. Reduced GSH was used as a standard for constructing the calibration line. The monitored molecules and the ions associated with them are as follows: gsh: 308. *m*/*z*, 613.2 *m*/*z*, 307.1 *m*/*z*, 615.2 *m*/*z*; pc2: 540.1 *m*/*z*, 538.1 *m*/*z*; pc3: 772.2 *m*/*z*, 770.2 *m*/*z*. Experiments were performed in triplicate and results were expressed as µM GSH equivalent/gDW. Statistical analysis was performed using one-way ANOVA with the Duncan post hoc test (*p* < 0.05), after the Shapiro–Wilk normality test and Levene’s homoscedasticity test using SPSS 25 software (IBM Corporation, Armonk, NY, USA).

## 4. Conclusions

This study provides the first detailed characterization of the SaPCS gene from *S. acutus*. Phylogenetic analysis placed SaPCS within the Scenedesmaceae, closely related to *Tetradesmus*/*Scenedesmus obliquus*, consistent with the presence of conserved diagnostic residues and genomic evidence for a single PCS locus in this clade.

Functional analyses indicate that SaPCS regulation is primarily linked to S nutrition rather than to Cr(VI) stress. RT-qPCR analysis revealed that transcriptional activation is strongly modulated by S availability, with divergent responses in wt vs. Cr-t strains, pointing to a role in S homeostasis and redox balance rather than in direct Cr(VI) detoxification.

Immunoblotting assays further identified two protein species: a ~70 kDa full-length form and a ~34 kDa truncated variant. Both forms were more responsive to S status than to Cr(VI) stress, and their higher basal levels in the tolerant strain suggest post-transcriptional and protein-level regulation linked to S metabolism. The ~34 kDa isoform likely corresponds to the catalytic N-terminal domain, arising from proteolytic processing or, more likely, alternative splicing. Indeed, three transcript variants (*SaPCSa*, *SaPCSb*, *SaPCSc*) with intron retention and/or exon skipping were identified, supporting the existence of truncated isoforms derived from the single *PCS* gene identified in *S. acutus* genome. Alternative splicing, non-AUG initiation, and translational readthrough may generate protein isoforms with distinct functional roles and subcellular localizations, potentially allowing the organism to fine-tune chelation capacity and adapt its stress responses. This mechanism could allow for the production of proteins with different physiological roles and involved in the response to different stresses, thus compensating for the presence of a single *PCS* gene in the genome.

However, mass spectrometry revealed that Cr(VI) treatment does not substantially enhance PC accumulation, with only minor, S-dependent variations. Overall, our data support a model in which Cr(VI) detoxification in *S. acutus* relies mainly on GSH-dependent conjugation and the antioxidant activity of the ascorbate–GSH cycle, while the production of PCS isoforms lacking the C-terminal domain contributes to coping with oxidative stress produced both by S-starvation and by Cr(VI) exposure. We propose that the transcriptional and translational plasticity of PCSs underlies physiological resilience without necessarily driving major PC output. This plasticity suggests that PCS expression is finely tuned at multiple levels, enabling organisms to balance thiol metabolism, redox status, and detoxification capacity according to environmental constraints.

Future priorities should focus on isoform-specific functional analyses, subcellular localization of truncated variants, and multi-omics integration to clarify how sulfur availability, redox state, and chromium metabolism intersect in *S. acutus*.

## Figures and Tables

**Table 1:** List of primers used for identification of *SaPCS* transcripts.

Primer	5′–3′ Sequence	Localization Within the Gene	Nucleotide Spanning on gDNA	Nucleotide Spanning on cDNA	Annealing Temperature
SaPCS Fmet	ATGCTGCCCAGCACACTGT	E1	1–20	1–20	60 °C
SaPCS F1	TTCAAGTAGTTTCTAGCCAGC	E1	80–100	80–100	60 °C
SaPCS F2	ATGAGCCCGCCTTTTGTGG	E3	1282–1300	446–464	60 °C
SaPCS R1	GGTGGCCTTGATGAAGTGC	E5	2892–2910	1017–1032	60 °C
SaPCS R2	CATGAGCAGCATGCGTGGC	E7	4996–5014	1919–1937	62 °C
SaPCS SplR1	CACACCCTCAGATATCTTCC	I4	2308–2327		60 °C
SaPCS SplR2	GTTGACGTATGGCAATGAGC	I4	2593–2611		60 °C
SaPCS SplR3	CGCCAGCTGCCATCTCGC	I5	3070–3087		62 °C

## Data Availability

The original contributions presented in this study are included in the article/Supplementary Material. Further inquiries can be directed to the corresponding author.

## Supplementary Materials

The following supporting information can be downloaded at https://www.mdpi.com/article/10.3390/plants15030510/s1, Figure S1: Multiple sequence alignment of PCS proteins; Figure S2: Sequence alignment of AthPCS1, AthPCS2 and SaPCS; Figure S3: Electrophoresis gel of end point PCR carried on with primers annealing in the 5′-terminal domain; Figure S4: Conceptual translation of the PCS variants found in *S. acutus*; Figure S5: Quantification of PC_2_ in the wild-type strain after 24 h exposure to Cd (2.25 µM) and Cr(VI) (1 mg/L) under S-sufficient condition. Table S1: PCS sequences belonging to different green algal taxa, retrieved from data banks and used for phylogenetic analysis; Table S2: PCS sequences retrieved from the Scenedesmaceae annotated genomes available in Phycocosm.

## Abbreviations

The following abbreviations are used in this manuscript:

AtPCS1	*Arabidopsis thaliana* Phytochelatin Synthase 1
BLAST	Basic Local Alignment Search Tool
CDS	coding DNA sequence
Cr(VI)	hexavalent chromium
Cr-t	chromium-tolerant strain
Cys	cysteine
GSH	glutathione
HM	heavy metal
LOEC	lowest observed effect concentration
PC	phytochelatin
PCS	phytochelatin synthase
PTC	premature termination codon
ROS	reactive oxygen species
RT-qPCR	Real-Time quantitative polymerase chain reaction
S	sulfur
SaPCS	*Scenedesmus acutus* phytochelatin synthase
*SaPCSa*/*SaPCSb*/*SaPCSc*	alternatively spliced *SaPCS* transcript variants
TIS	translation initiation site
UTR	untranslated region
wt	wild-type
