# Supplementary material for: Adaptive Plasticity of Phytochelatin Synthase Under Chromium Stress and Sulfur Availability in Scenedesmus acutus
Source: Plants (Basel). 2026 Feb 6;15(3):510. doi: 10.3390/plants15030510 (PMC12899622; doi:10.3390/plants15030510)
Supplement: Supplementary file 1 [file plants-15-00510-s001.zip › Supplementary materials/Supplementary Figures.pdf]

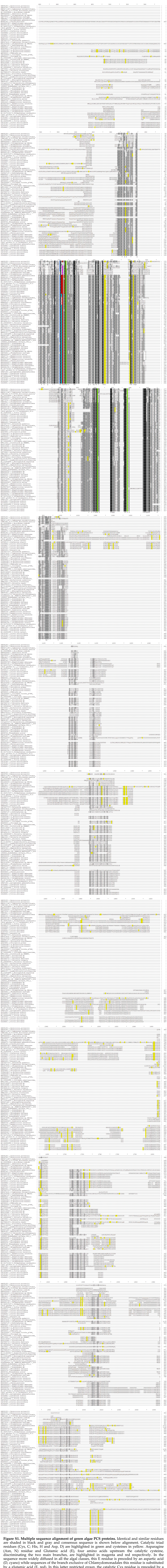

Figure S1. Multiple sequence alignment of green algae PCS proteins. Identical and similar residues are shaded in black and gray and consensus sequence is shown below alignment. Catalytic triad residues (Cys, C; His, H and Asp, D) are highlighted in green and cysteines in yellow. Asparagine (Asn, N) residue and Glutamic acid (E), four amino acids upstream the catalytic cysteine, discriminating the two main branches, are highlighted in magenta and red respectively. In the sequence more widely diffused in all the algal classes, this E residue is preceded by an aspartic acid (D, cyano) while sequences of the branch exclusive of Chlamydomonadales this residue is substituted by a glutamic acid (E, red). In this latter restricted group, the catalytic Cys residue is preceded by a glutamine (Q, orange) while in most of the remaining sequences this residue is represented by an alanine (Ala, A). In light blue the threonine (T) residue possible target of phosphorylation. Alignment was conducted with ClustalW and the accession no. of PCS protein sequences are reported in Table S1.

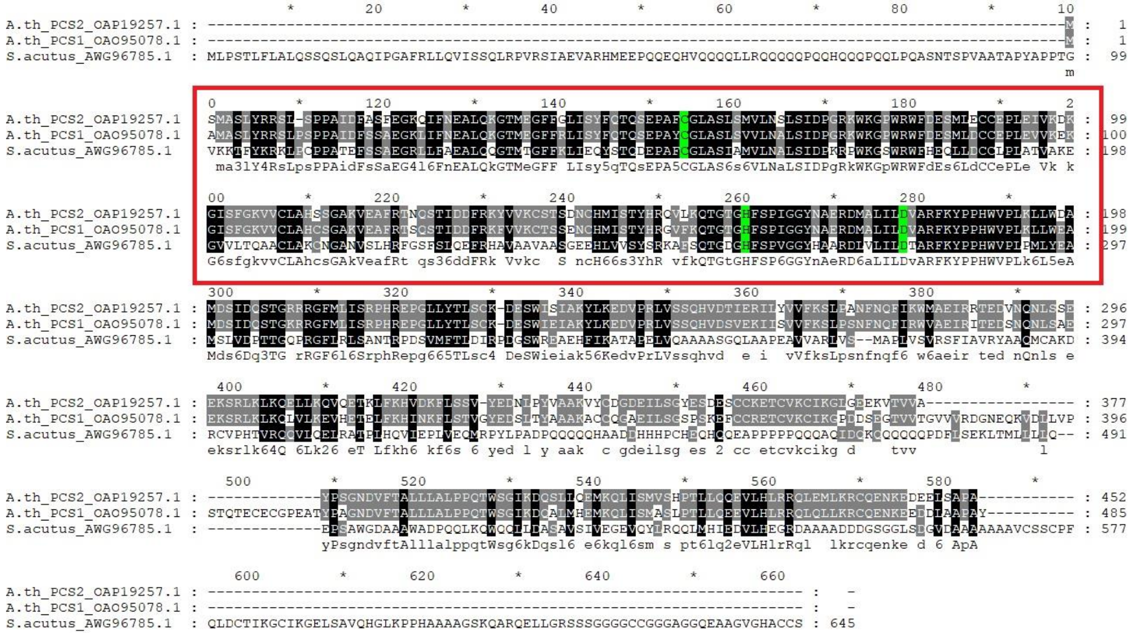

Figure S2. Sequence alignment of AthPCS1, AthPCS2 and SaPCS. Catalytic triad residues are highlighted in green, and conserved region is indicated by the red rectangle.

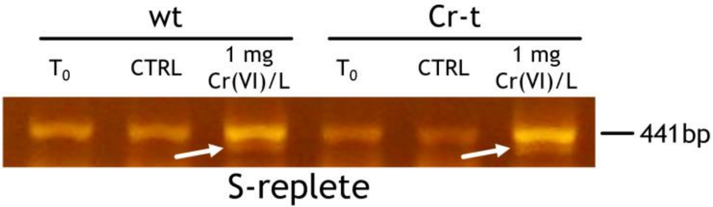

Figure S3. Electrophoresis gel of end point PCR carried on with primers annealing in the 5'-terminal domain. The weak band, shorter than expected (441 bp), in both the wt and the Cr-t strain exposed to 1 mg Cr(VI)/L in S-replete condition is indicate by the arrow.

>Sa_PCS
MLPSTLFLALQSSQSLQAQIPGAFRLLQVISSQLRPVRSIAEVARHMEEPQQEQHVQQQQLLRQQQQQPQQHQQQ
PQQLPQASNTSPVAATAPYAPPTGVKKTFYKRKLPCPPATEFSSAEGRLLFAEALQQGTMTGFFKLIEQYSTQDE
PAFCGLASIAMVLNALSIDPKRPWKGSWRWFHEQLLDCCLPLATVAKEGVVLTQAACLAKCNGANVSLHRFGSFS
LQEFRHAVAAVAASGEEHLVVSYSRKAFSQTGDGHFSPVGGYHAARDLVLILDTARFKYPPHWVPLPMLYEAMSL
VDPTTGQPRGFLRLSANTRPDSVMFTLDIRPDGSWREAEHFIKATAPELVQAAAASGQLAAPEAVVARLVSMAPL
VSVRSFIAVRYAAQMCAKDRCVPHTVRQQVLQELRATPLHQVIEPLVEQMRPYLPADPQQQQHAADDHHHPCHE
QHQQEAPPPPPQQQAQIDQKQQQQQPDFLSEKLTMLLLLQEPSAWGDAAAWADPQQLKQWQQLLDASAVSIVEG
EVQYLRQQLMHIEDVLHEGRDAAAADDDGSGGLSDGVDAAAAAAAVCSSCPFQLDCTIKGCIKGELSAVQHGLKP
PHAAAAGSKQARQELLGRSSSGGGGCCGGGAGGQEAAGVGHACCS

>SaPCSa
MLPSTLFLALQSSQSLQAQIPGAFRLLQVISSQLRPVRSIAEVARHMEEPQQEQHVQQQQLLRQQQQQPQQHQQQ
PQQLPQASNTSPVAATAPYAPPTGVKKTFYKRKLPCPPATEFSSAEGRLLFAEALQQGTMTGFFKLIEQYSTQDE
PAFCGLASIAMVLNALSIDPKRPWKGSWRWFHEQLLDCCLPLATVAKEGVVLTQAACLAKCNGANVSLHRFGSFS
VQEFRHAVAAVAASGEEHLVVSYIRKAFSQTGDGHFSPVGGYHAARDLVLILDTVRNEQTCVVCSVAIFDVKGMP
LQPCGVGRARWKISEGVHMVAALLLCAGCRACAL*HHAHYSALPSSLHPLLLPGCSHS*CLPLLLFAYQARFKYP
PHWVPLPMLYEAMSRVDPVARRPHHGAATLCSLPYV

>SaPCSb read on frame +1
MLPSTLFLALQSSQSLQAQIPGAFRLLQVISSQLRPVRSIAEVARHMEEPQQEQHVQQQQLLRQQQQQPQQHQQQ
PQQLPQASNTSPVAATAPYAPPTGVKKTFYKRKLPCPPATEFSSAEAPKMSPPFVGLPALPWCSMLCQLTPSGPG
RAAGAGSMSSCWTAACRWQQWQRRAWC*HRLPAWPSATAPTSRCTASAASACRSSGML*LLWLPVARSTWWSATA
ARHSARPATATSALLGDTMLRGTWC*S*TRYVMSRRVLCVALPYLMSRGCHFSPVGSAGRVGRYLRVCTW*LPCC
CVLAAGRVHCSTMLITLPFPHPSIPCCCPGVRTADAFPCSCLLIRRASSTRRTGCLFQCYMRPCRASTLSRVDPT
MGQPHCAHCHTSX

>SaPCSb read on Frame +3
AAQHTVPRAAVLAEPAGADTRRLSASSSNF*PASARAEHCRGRTAHGGATAGAARAAAAAAETATAATAAAPATA
AAATSGFQHFSCGSHSAVCPSYWSKENLLQAQVALPPCNRVQQCRSTQDEPAFCGLASIAMVLNALSIDPKRPWK
GSWRWFHEQLLDCCLPLATVAKEGVVLTQAACLAKCNGANVSLHRFGSFSLQEFRHAVAAVAASGEEHLVVSYSR
KAFSQTGDGHFSPVGGYHAARDLVLILDTVRNEQTCVVCSVAIFDVKGMPLQPCGVGRARWKISEGVHMVAALLL
CAGCRACAL*HHAHYSALPSSLHPLLLPGCSHS*CLPLLLFAYQARFKYPPHWVPLPMLYEAMSRVDPVARRPHH
GAATLCSLPYV

>SaPCSc
QVVSSQLRPVRSIAEVARHMEEPQQEQHVQQQQLLRQQQQQPQQHQQQPQQLPQASNTSPVAATAPYAPPTGVKK
TFYKRKLPCPPATEFSSAEGRLLFAEALQQGTMTGFFKLIEQYSTQDEPAFCGLASIAMVLNALSIDPKRPWKGS
WRWFHEQLLDCCLPLATVAKEGVVLTQAACLAKCNGANVSLHRFGSFSLQEFRHAVAAVAASGEEHLVVSYSRKA
FSQTGDGHFSPVGGYHAARDLVLILDTARFKYPPHWVPLPMLYEAMSLVDPTTGQPRGFLRLSANTRPDSVMFTL
DIRPDGSWREAEHFIKATAPELVQVGGCGSKHGVCCLWLSGDQLVTQLCPRFVVSLQASNINMRADSAGHQARWQ
LA*

Figure S4. Conceptual translation of the PCS variants found in *S. acutus*. Text in black: translation from exon sequences, text in green translation from intron sequences. Highlighted in green: catalytic triad; highlighted in red and yellow: end and start of two consecutive exons; highlighted in cyano: stops produced by the Premature Termination Codons. Highlighted in grey the putative mitochondrial signal peptide. Highlighted in magenta the threonine and arginine residue necessary for the second substrate pocket formation.

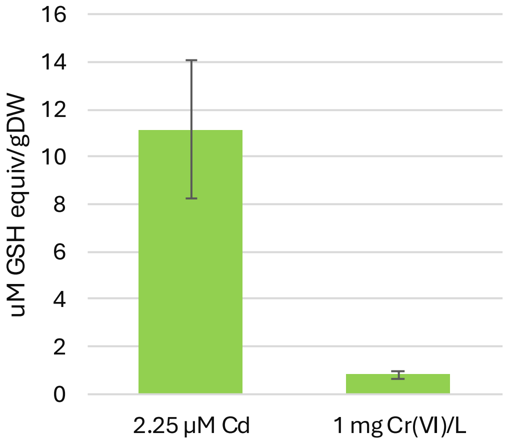

Figure S5. Quantification of PC_2 in the wild-type strain after 24 h exposure to Cd (2.25 µM) and Cr(VI) (1 mg/L) under S-sufficient condition. The results were reported as the mean value (± standard deviation) of three biological replicates.
